# Functions and Signaling Pathways of Amino Acids in Intestinal Inflammation

**DOI:** 10.1155/2018/9171905

**Published:** 2018-02-26

**Authors:** Fang He, Chenlu Wu, Pan Li, Nengzhang Li, Dong Zhang, Quoqiang Zhu, Wenkai Ren, Yuanyi Peng

**Affiliations:** ^1^College of Animal Science and Technology, Southwest University, Chongqing 400716, China; ^2^College of Animal Science, South China Agricultural University, Guangzhou 510642, China; ^3^College of Veterinary Medicine, Yangzhou University, 48 Wenhui East Road, Yangzhou, Jiangsu 225009, China

## Abstract

Intestine is always exposed to external environment and intestinal microorganism; thus it is more sensitive to dysfunction and dysbiosis, leading to intestinal inflammation, such as inflammatory bowel disease (IBD), irritable bowel syndrome (IBS), and diarrhea. An increasing number of studies indicate that dietary amino acids play significant roles in preventing and treating intestinal inflammation. The review aims to summarize the functions and signaling mechanisms of amino acids in intestinal inflammation. Amino acids, including essential amino acids (EAAs), conditionally essential amino acids (CEAAs), and nonessential amino acids (NEAAs), improve the functions of intestinal barrier and expressions of anti-inflammatory cytokines and tight junction proteins but decrease oxidative stress and the apoptosis of enterocytes as well as the expressions of proinflammatory cytokines in the intestinal inflammation. The functions of amino acids are associated with various signaling pathways, including mechanistic target of rapamycin (mTOR), inducible nitric oxide synthase (iNOS), calcium-sensing receptor (CaSR), nuclear factor-kappa-B (NF-*κ*B), mitogen-activated protein kinase (MAPK), nuclear erythroid-related factor 2 (Nrf2), general controlled nonrepressed kinase 2 (GCN2), and angiotensin-converting enzyme 2 (ACE2).

## 1. Introduction

The intestine is supreme digestive organ of humans and numerous animals including the small intestine and large intestine. Small intestine covers the ileum, jejunum, and duodenum, while large intestine includes the colon, cecum, and rectum [1]. Intestinal tract has numerous functions, including digestion and absorption of nutrients, recognition of external factors, and transduction of signaling concerned with innate and adaptive immunity [2]. Continuing to experience external stressors (e.g., dietary ingredients, intestinal microorganism, and environmental factors), easily, leads to intestinal damage and dysfunction [3]. Thus the intestine is usually in a situation of inflammation, which is related to certain illness, including diarrhea, inflammatory bowel disease (IBD), and irritable bowel syndrome (IBS) [4]. IBD comprise Crohn's disease (CD) and ulcerative colitis (UC) [5]. CD is normally located in whole intestine, influencing primarily intestinal wall [6]. UC is limited in the colon as well as rectum, affecting mainly the mucosal layer [7]. The production of proinflammatory cytokines, including interleukin-1 (IL-1), IL-6, IL-17, IL-22, IL-23, tumor necrosis factor-*α* (TNF-*α*), and interferon-*γ* (IFN-*γ*), highly shapes the development of IBD [8].

The metabolic profiling of amino acid in UC differs from control group, which indicates certain amino acids would be novel biomarkers for early diagnosis and treatment of patients with UC [9]. For example, the levels of glutamine (Gln), glutamate (Glu), methionine (Met), tryptophan (Trp), and histidine (His) are significantly lower in UC patients than in the normal control group, but asparagine (Asp) and isoleucine (Ile) are quite the reverse. Recent studies also show that amino acids have significant roles in the intestinal inflammation. For example, Trp of essential amino acids (EAAs) exerts beneficial regulatory function in mucosal growth or maintenance and alleviation of intestinal inflammation by 5-hydroxytryptophan (5-HT) signaling pathway [10], in the recovery of colitis by caspase recruitment domain family member 9 (Card9) [11, 12], and in the function of intestinal homeostasis and anti-inflammation by aryl hydrocarbon receptor (AHR) ligands in the intestine [13, 14]. Gln, one of nonessential amino acids (NEAAs), regulates anti-inflammatory effects dependent on its function by intestinal tight junctions (TJ), mechanistic target of rapamycin (mTOR), mitogen-activated protein kinase (MAPKs), and nuclear factor-kappa-B (NF-*κ*B) signaling pathways [15–18]. Arg is a conditionally essential amino acid (CEAAs) and has a critical function in treating intestinal inflammation by manipulation of immune responses, oxidative system, and intestinal metabolism [6, 19, 20]. Leu is a member of branched chain amino acids (BCAAs), and its deprivation may ameliorate colitis and intestinal inflammation via the amino acid sensor general controlled nonrepressed kinase (GCN2) [21, 22]. Aromatic amino acids (AAAs), including Trp, Phe, and Tyr, attenuate intestinal inflammation through activating calcium-sensing receptor (CaSR) in piglets [23]. The review aims to summarize the roles and molecular mechanisms of amino acids in the intestinal inflammation.

## 2. Amino Acids and Intestinal Inflammation

According to nutrition demand, amino acids are traditionally divided into 8 kinds of EAAs, 10 kinds of NEAAs, and 2 kinds of CEAAs. EAAs are only acquired from the nutrient by amino acid transporters, such as Trp, Leu, and Phe. NEAAs can be synthesized via certain elements* in vivo* (e.g., Glu, Gly, and Ser) [24]. There are two types of special amino acids, as they are neither the EAAs, nor the NEAAs, including Arg and His, which are EAAs for infants but not for adults; thus they are named as CEAAs. The protective functions of amino acids in the intestine may be closely connected with the apoptosis and proliferation of intestinal epithelial cells (IECs), expression of tight junction proteins (TJPs), alleviation of intestinal inflammation and oxidative stress by inhibiting NF-*κ*B signaling pathway, and activating nuclear erythroid-related factor 2 (Nrf2) signaling pathway [25, 26]. NF-*κ*B and Nrf2 are two critical signaling pathways that are related to inflammation and oxidation. NF-*κ*B upregulates expressions of various proinflammatory cytokines (e.g., IL-1*β*, IL-6, IL-8, and TNF-*α*) [27]. Nrf2 suppresses the production of proinflammatory cytokines and increases the expressions of antioxidative genes [28, 29]. The oxidative stress and inflammatory mediators are the main etiological factors in IBD; hence, amino acids are expected to alleviate it as the antioxidants and anti-inflammatory agents [30, 31]. For example, gamma aminobutyric acids (GABA) signaling negatively regulates the production of proinflammatory factors via inhibiting the activation of NF-*κ*B pathway; thus it shows various advantageous functions in the progression of IBD [32]. Another characteristic of IBD is to destroy the integrity of intestinal epithelial barrier (IEB) [33], which regulates the absorption of nutrition and restricts the entry of pathogens, composed of topmost TJs, bottom adherent junctions (AJs), and desmosomes [34]. The function of the IEB is determined by TJs, a protein complex, including occludin, claudin family, and junctional adhesion molecules (JAMs) [35, 36], and amino acids have critical roles in the expression of TJPs [37, 38]. For example, Trp enhances the expression of occluden-1, occluden-2, occludin, claudin-3, and claudin-4 in the intestine of pig [39, 40]. Arg and Glu supplementation improve permeability and TJs protein expression [41, 42]. Besides, the protective effect of amino acids is also associated with endoplasmic reticulum (ER) stress and autophagy [43]. Abundant ER stress leads to apoptosis [44] and is a critical factor for intestinal barrier integrity and intestinal homeostasis [45]. Autophagy regulated by mTOR signaling is crucial for inhibiting intestinal inflammation and maintaining intestinal homeostasis [46]. The mTOR signaling has momentous functions in cell proliferation, differentiation, growth, and metabolism [47, 48]; thus it may be a target for the therapy of intestinal inflammation. Furthermore, MAPK signaling is another important signaling pathway for amino acids and intestinal inflammation. The MAPK signaling of mammals is mainly composed of MAPKS extracellular signal-regulated kinase (ERK), the c-Jun N-terminal kinase (JNK), and p38 MAPK pathways, which play important roles in cell growth, proliferation, differentiation, migration, inflammation, and survival, and is associated with pathogenesis of several human diseases, including IBD [49–52]. Some amino acids have critical roles in the activation of MAPK pathway [53, 54]. For example, Asn improves intestinal integrity by downregulating intestinal proinflammatory cytokine through MAPKp38 and decreases enterocyte apoptosis via MAPKp38 and ERK1/2 [55]. Arg alleviates LPS induced immune damage in fish intestine and the enterocytes by downregulating MAPKp38 [56]. Gln combined with Arg decreases the production of TNF-*α* and other proinflammatory cytokines probably through its regulation in MAPKp38 [57].

### 2.1. EAAs and Intestinal Inflammation

EAAs have significant effects in intestinal inflammation. It is reported that Phe possesses beneficial effects in the treatment of IBD by inhibiting TNF-*α* productions and enhancing immune responses [63]. Phe with chromium has a protective effect against IBD induced by indomethacin in rats, which might be attributed to antioxidant and anti-inflammatory characteristics of Phe [30]. Phe regulates intestinal hormone release as well as glucose tolerance and inhibits food intake of rodents by CaSR, which may be a potential therapy for obesity and diabetes [64]. Met is able to modulate metabolism, innate immunity, and digestion of mammals and generate glutathione to neutralize oxidative stress [112]. Met inhibits the increase of paracellular permeability mediated by TNF-*α*, which may be related to antioxidant metabolites (e.g., taurine and glutathione) to improve intestinal homeostasis [65]. Abundant Met is crucial for intestinal integrity and intestinal antioxidant capacity [66]. Lys influences the digestion of food and the expressions of amino acid transporters in the intestine [68]. Poly-L-lysine (PL) is a homopolymer of L-lysine and reduces the production of IL-8 in the IECs induced by TNF-*α*; thus, PL supplementation inhibits the expressions of proinflammatory cytokines by activating CaSR in the intestine [69]. Glucose-lysine Maillard reaction products (Glc-Lys MRPs) ameliorate DSS-induced colitis, increase glutathione content as well as antioxidant activities, and suppress the inflammatory cytokines and NF-*κ*B [70, 71]; thus they can be used for preventing or treating IBD. Thr is a primary ingredient of intestinal IgA and mucins; thus, malnutrition of Thr induces inflammation and affects the immune responses through the NF-*κ*B pathway [72]. Dietary supplementation with Thr has a favorable regulatory function on the intestinal barrier and immunity of broiler chicks infected with* Eimeria maxima* [73]. Thr insufficiency impairs intestinal immune response and increases inflammation associating with NF-*κ*B and mTOR pathways in young grass carp infected by* Aeromonas hydrophila* [74]. BCAAs (e.g., Leu, Val, and Ile) enhance intestinal immune defense system through improving morphological integrity and immunoglobulin production in the intestine [113]. Leu enhances cell proliferation and the expressions of amino acid transporters by the activation of mTOR [77, 78]. However, high concentration of BCAAs increases oxidative stress and inflammation by mTOR and NF-*κ*B [114]; thus, diets with low Leu ameliorate symptoms of colitis and intestinal inflammation via the amino acid sensor GCN2 in colitis model [22]. Ile induces the expression of *β*-defensins via G-protein-coupling receptors (GPCRs) and ERK/MAPK signaling pathways [79]. And a recent study found that dietary Ile improves intestinal immune function, antioxidant capacity, and microbial population and regulates gene expression of antioxidant enzyme, tight junctions, Nrf2, p38, and ERK1 in the intestine of Jian carp [115]. The research of Val in intestinal inflammation is relatively rare, but *γ*-glutamyl Val diminishes inflammation in colitis via CaSR signaling and inhibits TNF-*α* pathways in IECs [76]. Moreover, Trp, Phe, and Tyr possess aromatic nucleus so they are named as aromatic amino acids (AAAs), which reduce intestinal inflammation by activating CaSR in piglets [23]. The CaSR is one of the GPCRs, which participates in nutrient sensing and ion homeostasis maintaining, hormone and fluid secretion, cell differentiation, and apoptosis in the intestine [116, 117]. The deficiency of epithelial CaSR leads to weak intestinal integrity, alteration of microbiota composition, and acceleration of proinflammatory immune responses [118]. The gene expression of CaSR may be regulated by vitamin D, extracellular Ca^2+^, and cytokines [119–123]. However, L-amino acids such as L-Ala, L-Phe, and L-Trp are the agonists of the CaSR; thus they are effective in preventing and treating IBD and other diarrheal diseases via CaSR [123–127]. CaSR activated by Trp exerts anti-inflammation roles via activating the complex of Β-arrestin 2 (*β*-arr2) and TAK1-binding protein 1 (TAB1) to inhibit NF-*κ*B and MAPK pathway in IECs [58].

**Figure 1:**
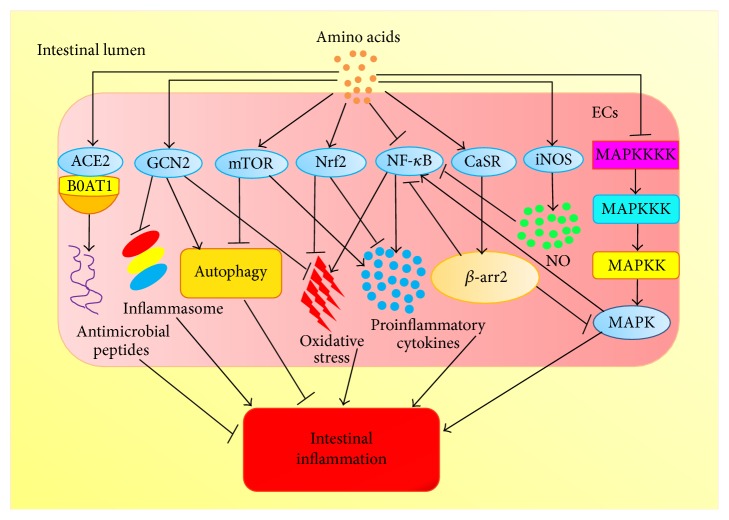
Possible signaling mechanisms of amino acids in intestinal inflammation in the ECs are illustrated. Amino acids ameliorate intestinal inflammation by impressing NF-*κ*B and MAPK pathway. Amino acids activate Nrf2 pathway to regulate intestinal inflammation via inhibiting oxidative stress and the expressions of proinflammatory cytokines. Amino acids activate iNOS to inhibit NF-*κ*B pathway by the production of NO. ACE2 combines B0AT1 to regulate uptake of Trp in IECs, which activates expressions of antimicrobial peptides to regulate intestinal microbiota. ACE2 illuminate intestinal inflammation by regulating innate immune responses and intestinal microbiota is not shown in the figure. GCN2 regulates intestinal inflammation by inhibiting inflammasome activation, triggering autophagy, and preventing oxidative stress. CaSR activated by Trp exerts anti-inflammation roles via activating the complex of Β-arrestin 2 (*β*-arr2) to inhibit NF-*κ*B and MAPK pathway in IECs. After being activated by amino acids, mTOR signaling could inhibit autophagy.

**Figure 2:**
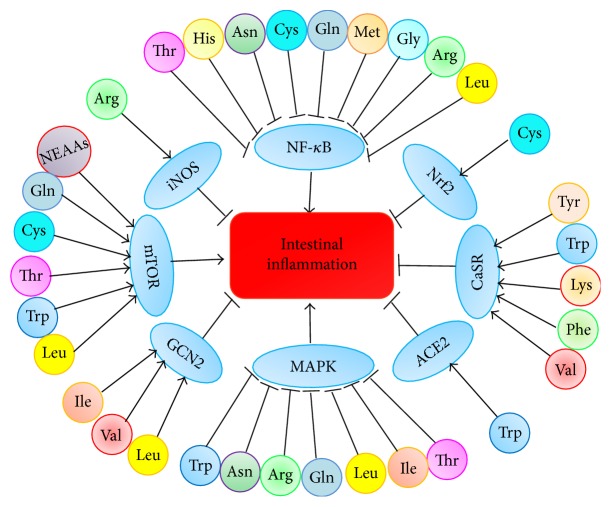
Specific signaling pathways of different amino acids in intestinal inflammation are showed. Thr, His, Arg, Leu, Asn, Cys, Gln, Met, and Gly inhibit NF-*κ*B signaling pathway to ameliorate intestinal inflammation. Thr, Arg, Leu, Asn, Trp, Gln, and Ile inhibit MAPK signaling pathway to relieve intestinal inflammation; Leu, Ile, and Val activate GCN2 pathway to improve intestinal inflammation. Tyr, Lys, Trp, Val, and Phe activate CaSR pathway to attenuate intestinal inflammation. NEAAs, Thr, Gln, Leu, Cys, and Trp may promote intestinal inflammation through activating mTOR. Trp reduces intestinal inflammation via activating ACE2 pathway; Cys decreases intestinal inflammation through activating Nrf2 signaling pathway. Arg decreases intestinal inflammation by iNOS signaling pathway.

The best example for EAAs in intestinal inflammation comes from Trp. Trp has a vital role in intestinal inflammation via 5-HT signaling pathway [59]. 5-HT signaling is made up of tryptophan hydroxylase-1 (TPH-1), 5-HT receptors, and serotonin reuptake transporter (SERT) [128]. Intestinal mucosa is the prime position of 5-HT synthesis catalyzed by TPH-1 [129]. Released from enterochromaffin cells, 5-HT starts to play its regulative role in the intestine (e.g., intestinal motility, fluid secretion) [130]. Functions of 5-HT are excised through a variety of 5-HT receptors; thus, 5-HT3 receptor and 5-HT4 receptor are principally associated with IBS [131]. The 5-HT3 receptor is only a ligand-gated ion channel, and recent evidences demonstrated that 5-HT3 receptor antagonists exert anti-inflammatory functions via inhibiting the production of inflammatory cytokines in colitis [132]. 5-HT2B is one of the 5-HT2 receptors, which plays vital parts in IBS and has a remarkable effect in the human colon [133, 134]. 5-HT7 receptor, a member of the GPCRs, whose expression in IBS is upregulated [135], regulates the severity of intestinal inflammation in colitis or CD [14]. Evidence indicates that the expression of IL-10 receptor is regulated by AHR in the colon [136]. Mice lacking IL-10 or IL-10R are sensitive to colitis [137] because IL-10 is a significant anti-inflammatory cytokine that represses the production of proinflammatory mediators. Kynurenine (Kyn) from Trp metabolism binds to AHR to regulate systemic inflammation, and research found that levels of Kyn are increased during intestinal inflammation to induce the expression of IL-10R [138, 139]. Moreover, dietary Trp alleviates SDS-induced colitis by AHR in mice [60]. AHR contributes to the expressions of IL-22 and the development of T-helper type 17 (Th17) cells [140]. IL-22 has significant functions in maintaining intestinal homeostasis [141]. The metabolism of Trp modulates the production of IL-22 by AHR [13]. Furthermore, a recent research indicates that Card9, a susceptibility gene of IBD, promotes the recovery of colitis by metabolizing Trp into AHR ligands to activate IL-22 signaling pathway in innate immune response [11]. The supplementation of Trp inhibits Th1 differentiation in vivo [61]. And L-Trp supplementation decreases the destruction of intestinal barrier triggered by stress via modulating 5-HT metabolism in broilers [62]. Collectively, EAAs mainly exert anti-inflammatory roles by NF-*κ*B, CaSR, MAPK, and mTOR signaling pathway to restrain the expressions of proinflammatory cytokines. The functions and signaling pathways of EAAs in the intestinal inflammation are showed in Table 1. Possible signaling mechanisms of EAAs on the intestinal inflammation in the ECs are showed in Figure 1. Specific signaling pathways of EAAs in intestinal inflammation are showed Figure 2.

### 2.2. CEAAs and Intestinal Inflammation

Arg plays crucial roles in regulating intestinal inflammation via immune response, oxidative system, tight junction, and intestinal metabolism [142]. Arg as a nutritional supplement reduces the expressions of IL-1*β* and IL-6, as well as delaying the onset of colitis when the colitis is not very serious, and inhibits the increase of intestinal epithelial permeability by preventing inflammatory neutrophil recruitment and oxidative stress in the DSS-induced colitis [31]. Besides, Arg reduces the activation of IL-1*β*-induced NF-*κ*B signaling pathway [80]. Nitric oxide (NO) also inhibits the activation of NF-*κ*B signaling [143], and Arg decreases production of IL-8 during the intestinal inflammation which may occur through increasing the production of NO via inducible nitric oxide synthase (iNOS) [144]. L-Arg improves survival rate as well as antineoplastic properties and regulates the metabolism of T cells [81]. Our previous reports indicated that dietary supplementation of Arg partly alters the progression of porcine circovirus type 2 (PCV2) infection [82]. Dietary supplementation of Arg has significant influence in colitis treated with dextran sulfate sodium (DSS) via NF-*κ*B signaling pathways [83]. Arg supplementation increases immune responses, growth characteristics, and morphology of small intestine in weaned piglets [84]. Arg supplementation changes the intestinal microbiota, which is conducive to activate intestinal innate immune responses by NF-*κ*B signaling pathway [145]. His is another CEAAs and an important anti-inflammatory factor, which inhibits the production of IL-8 induced by oxidative stress or TNF-*α* through controlling the activation of NF-*κ*B in the IECs [3]. His supplement alleviates colitis of murine by suppressing the generation of proinflammatory mediators; thus it may have therapeutic utility for CD by inhibiting the activation of NF-*κ*B [85]. Moreover, the decrease of His increases relapsing risk in the emission of UC patients; thus His may be a noninvasive predictive marker in the intestinal inflammation [86]. Thus taking advantage of Arg or His supplementation to prevent or treat intestinal inflammation is a kind of new adjuvant treatment strategy for intestinal diseases associating with inflammation. The functions and signaling pathways of CEAAs in the intestinal inflammation are showed in Table 1. In conclusion, CEAAs play a critical anti-inflammatory role in the intestine through its regulatory functions in immune responses, NF-*κ*B pathway. Possible signaling mechanisms of CEAAs on the intestinal inflammation in the ECs are showed in Figure 1. Specific signaling pathways of CEAAs in intestinal inflammation are showed Figure 2.

### 2.3. NEAAs and Intestinal Inflammation

NEAAs play beneficial roles in the intestinal inflammation. The deficiency of NEAAs damages intestinal barrier and expressions of TJPs (e.g., claudin-1, ZO-1) in IECs, which triggers protective autophagy via mTOR pathway [146]. Cys supplementation suppresses intestinal inflammation through increasing the expressions of TJPs and decreasing the expressions of proinflammatory factors in colitis [45]. Cys exerts protective functions in the intestinal barrier that involves anti-inflammation and antioxidation by suppressing the NF-*κ*B pathway and activating the Nrf2 signaling pathway [25]. N-Acetylcysteine (NAC) protects intestinal barrier in piglets induced by LPS via mTOR, NF-*κ*B, and MAPK signaling pathway [95, 96]. Accumulating evidence indicates that Gly enhances intestinal mucosal barrier and inhibits oxidative stress via suppressing the activation of NF-*κ*B and the production of TNF-*α*, IL-1, and IL-6 [97–101]. Several lines of evidence have indicated that dietary supplementation of Glu has significant roles in the proliferation of IECs, the function of mucosal barrier, and the increase of antioxidative capacity to control intestinal permeability and decrease proinflammatory cytokines production [102, 103]. Glu effectively regulates oxidative stress and intestinal injury in piglets treated with the mycotoxin deoxynivalenol (DNO) [104]. Pro supplementation has crucial roles in regulating the proliferation and differentiation of IECs, increasing superoxide dismutase (SOD) activities, and expressions of TJPs [105, 106]. Dietary supplementation of Pro exerts advantageous immune-stimulatory functions in the mice immunized with inactivated* Pasteurella multocida* (Pm) [107]. Asp or Asn has important functions in stimulating the proliferation of IECs and triggering immune response to attenuate intestinal injury and restore intestinal morphology as well as barrier function impaired with lipopolysaccharide (LPS) via inhibiting NF-*κ*B signaling pathway [55, 108, 109]. Dietary supplementation of Asp alleviates growth suppression and oxidative stress of piglets treated by H_2_O_2_ [110]. Ser promotes the synthesis of mucins and improves the composition of gut microbiota in the rats induced by DSS [75]. Ser immediately regulates adaptive immunity via modulating T cell proliferation [111]. Tyr and alanine (Ala) are necessary ingredients of protein synthesis and immunity, which also have advantageous functions in the intestinal inflammation [63]. However, their molecular mechanism and signaling pathways are still unclear; thus further numerous investigations are needed to be done to address these issues.

Gln, the richest amino acid in plasma, plays an important role in maintaining the integrity of intestinal barrier. Studies showed that deficiency of Gln can lead to villus atrophy, reduction in expression of TJPs, and increase in permeability of intestine, but Gln supplement can improve gut barrier function in IBS [87]. A lot of evidence shows that Gln plays an anti-inflammatory role by affecting the NF-*κ*B as well as STAT signaling pathways [88]. I*κ*B proteins are phosphorylated by I*κ*B kinase to release NF-*κ*B to activate the immune responses. Activated NF-*κ*B complex triggers the expressions of IL-6 and TNF-*α*, which activates T cells and antigen-presenting cells (APCs) [147]. Gln inhibits NF-*κ*B pathway by increasing the expression of heat shock proteins (HSPs) mediated by HSF-1 to suppress the expressions of inflammatory cytokines [17, 89]. STAT proteins are transcription factors regulating intestinal inflammation by mediating the expression of IL-6 [148]. Gln influences the activation of STAT signaling that was proved by reducing the phosphorylation of STAT1 as well as STAT5 [90]. When Gln is deficient, the expression of STAT4 is increased; nevertheless, the expression of STAT4 and IL-8 is reduced after supplementing Gln [149]. From the above studies, Gln may play anti-inflammatory role via preventing the activity of STAT and NF-*κ*B to regulate the production of IL-6 as well as IL-8 in the intestinal inflammation. Moreover, Gln has protective effects in colitis by mTOR signaling pathway [91]. Deficiency of Gln triggers autophagy and hinders amino acid metabolism in IECs by inactivating mTOR and MAPK/ERK signaling pathways, but Gln supplementation recovers the phenomenon [18]. And another study found that the growth of enterocyte is induced by Gln via mTOR without AMPK signaling pathway [92]. Furthermore, Gln affects the production of intestinal SIgA to protect IECs from harmful factors [93]. The supplementation of dietary Gln may suppress intestinal* enterotoxigenic Escherichia coli* infection by innate immunity [94]. Collectively, NEAAs exert anti-inflammatory roles associating with NF-*κ*B, MAPK, mTOR, and Nrf2 pathways. The functions and signaling pathway of NEAAs in the intestinal inflammation are showed in Table 2. Possible signaling mechanisms of amino acids on the intestinal inflammation in the ECs are showed in Figure 1. Specific signaling pathways of CEAAs in intestinal inflammation are showed in Figure 2.

## 3. Amino Acid Sensor GCN2 Regulates Intestinal Inflammation

GCN2 is a key sensor of integrated stress response (ISR) and can sense amino acid depletion [54, 150]. Previous, study reported that GCN2 deficient mice cannot effectively deal with the starvation of EAAs, leading to change in nutrition intake and increase in death [151]. A recent study found that GCN2 deficiency increases intestinal inflammation in IECs as well as APCs and Th17 cells responses in colitis [22]. Thus intestinal inflammation may be associated with amino acid sensing pathway GCN2, which may suppress intestinal inflammation by inhibiting inflammasome activation, triggering autophagy, and preventing oxidative stress and Th17 cells differentiation in colitis [22]. When amino acids are insufficient, the homeostasis of amino acids is recovered by arresting translational after the translation initiator eukaryotic initiation factor 2 (eIF2) phosphorylated by GCN2 [54, 152]. When amino acids are redundant, amino acids could lead to intestinal inflammation on account of lacking GCN2 stimulation [21]. Another research indicates that GCN2 is essential for regulating the expressions of inflammatory cytokines and immune responses in myeloid cells [153]. Therefore, GCN2 may be a fine target to manage inflammatory illness.

## 4. ACE2 Connects Amino Acid Malnutrition and Intestinal Inflammation

A series of evidence suggests that malnutrition is related to intestinal inflammation [154]. A study indicates that amino acid malnutrition is always related to intestinal inflammation via angiotensin-converting enzyme 2 (ACE2), which plays significant roles in amino acids homeostasis, innate immune responses, and intestinal microbiota [155]. ACE2 is an important enzyme of renin-angiotensin system (angiotensin 1–7), which is expressed on various organs including small intestine, and has a crucial function in controlling intestinal inflammation as a stabilizer of neutral amino acid transporters [156]. Angiotensin 1–7 treatment has an anti-inflammatory effect on IBD by reducing the activity of MAPK and NF-*κ*B [157]. ACE2 regulates innate immune response and intestinal microbiota, which illuminates intestinal inflammation under conditions of severe malnutrition [158]. Mice with ACE2 knockout and ACE2 mutation show the decline in the uptake of Trp, leading to the decrease of expressions of antimicrobial peptides and the change of intestinal microbiota, resulting in the high sensitivity to intestinal inflammation, which is restored by Trp supplementation [155]. The acquisition and uptake of Trp primarily rely on B0AT1, whose expression is provoked by ACE2 in the IECs [159]. ACE2 provides a new way for the therapy of intestinal inflammation.

## 5. Conclusion

In conclusion, the functions of amino acids in intestinal inflammation are mainly associated with improving intestinal barrier, attenuating intestinal injury, suppressing oxidative stress, and inhibiting the expressions of proinflammatory cytokines. These functions are finished by a series of signaling mechanisms, including NF-*κ*B, MAPK, Nrf2, mTOR, iNOS, CaSR, ACE2, and GCN2. However, the exact molecular mechanism of some amino acids is not unclear, such as Ala and Ser. Therefore, there is still much work to be done to explore the relevant signaling pathways. Future studies also should concentrate on the functions and signaling pathways of amino acids to explore safe and effective therapeutic schedule for human and animals in the intestinal inflammation.

## Figures and Tables

**Table 1:** The functions and signaling pathways of EAAs and CEAAs in intestinal inflammation.

Amino acids	Functions	Signaling pathways	References
Tryptophan	↑IL-22, intestinal barrier ↓Il-1*β*, Il-6, IL-8, TNF-*α*, Th1 cells	5-HT, mTOR, AHR Card9, ACE2, CaSR, MAPK	[11, 13, 58–62]

Phenylalanine	↑anti-inflammatory ability, GSH ↓TNF-*α*, IL-6, IL-8, oxidative stress	CaSR	[30, 63, 64]

Methionine	↑intestinal integrity, Cys and GSH ↓IL-1*β*, TNF-*α*, oxidative stress	NF-*κ*B	[65–67]

Lysine	↑GSH, SOD, CAT ↓IL-1*β*, IL-6, IL-17, TNF-*α*, INF-*γ*	CaSR, NF-*κ*B	[68–71]

Threonine	↑MUC2, IgA, intestinal barrier	NF-*κ*B, mTOR, MAPK	[72–75]

Valine	↑immunoglobulin production ↓TNF-*α*, IL-6, INF-*γ*, IL-1*β*, and IL-17	GCN2, CaSR	[22, 76]

Leucine	↑intestinal integrity ↓intestinal inflammation	mTOR, GCN2 NF-*κ*B, MAPK	[22, 77, 78]

Isoleucine	↑expressions of *β*-defensins	GCN2, GPCRs, MAPK	[22, 79]

Arginine	↑regulation of intestinal microbiota ↓oxidative stress, IL-1*β* and IL-6 ↓inflammatory neutrophil recruitment	NF-*κ*B iNOS MAPK	[31, 80–84]

Histidine	↓IL-6, IL-8, TNF-*α*	NF-*κ*B	[3, 85, 86]

Functions of EAAs and CEAAs in intestinal inflammation mainly depend on NF-*κ*B, iNOS, MAPK, ACE2, GCN2, CaSR, and mTOR signaling pathways. AHR: aryl hydrocarbon receptor; 5-HT: 5-hydroxytryptophan; Card 9: caspase recruitment domain family member 9; mTOR: mechanistic target of rapamycin; MUC2: mucin 2; MPO: myeloperoxidase; CaSR: calcium-sensing receptor; MAPK: mitogen-activated protein kinase; NF-*κ*B: nuclear factor-kappa-B; ROS: reactive oxygen species; Cys: cysteine; GSH: glutathione; iNOS: inducible nitric oxide synthase; ACE2: angiotensin-converting enzyme 2; GPCRs: G protein-coupled receptors; SOD: superoxide dismutase; CAT: catalase; GCN2: general controlled nonrepressed kinase 2.

**Table 2:** The functions and signaling pathways of NEAAs in intestinal inflammation.

Amino acids	Functions	Signaling pathways	References
Glutamine	↑intestinal barrier, anti-inflammation, IgA ↓proinflammatory cytokines	NF-*κ*B, mTOR MAPK/ERK	[87–94]
Cysteine	↑tight junctions, intestinal barrier, and homeostasis ↓TNF-*α*, IL-1*β*, IL-6, and IL-8, oxidative stress	NF-*κ*B, Nrf2 mTOR	[25, 45, 95, 96]
Glycine	↑intestinal mucosal barrier ↓TNF-a, IL-1, and IL6, oxidative stress	NF-*κ*B	[97–101]
Glutamate	↑intestinal mucosal barrier ↓TNF-*α*, IL-1 and oxidative stress	Unclear	[102–104]
Proline	↑SOD, tight junction proteins	Unclear	[75, 105–107]
Aspartate/ asparagine	↑intestinal barrier function ↓proinflammatory cytokines	NF-*κ*B MAPK	[55, 108–110]
Tyrosine	↑intestinal health and immune function	CaSR	[63]
Alanine	↑intestinal defense and protection function	Unclear	[63]
Serine	↑colonic protection, mucosal healing ↑mucin synthesis, gut microbiota	Unclear	[75, 111]

Functions of NEAAs in intestinal inflammation mainly rely on NF-*κ*B, Nrf2, MAPK, mTOR, and CaSR signaling pathways. NF-*κ*B: nuclear factor-kappa-B; CaSR: calcium-sensing receptor; mTOR: mechanistic target of rapamycin; MAPK: mitogen-activated protein kinase; Nrf2: transcription factor NF-E2-related factor 2; SOD: superoxide dismutase.

## Abbreviations

Arg: Arginine
Ala: Alanine
Asp: Aspartate
Asn: Asparagine
AHR: Aryl hydrocarbon receptor
AAAs: Aromatic amino acids
APCs: Antigen-presenting cells
AJPAS: Adherent junction proteins
BCAAs: Branched chain amino acids
CEAAs: Conditionally essential amino acids
CD: Crohn's disease
Card9: Caspase recruitment domain family member 9
CaSR: Calcium-sensing receptor
Cys: Cysteine
ER: Endoplasmic reticulum
eIF2: Eukaryotic initiation factor 2
EAAs: Essential amino acids
Glu: Glutamate
Gln: Glutamine
Gly: Glycine
GCN2: General controlled nonrepressed 2 kinase
GPCRs: G-protein-coupling receptors
His: Histidine
HSPs: Heat shock proteins
IBD: Inflammatory bowel disease
IBS: Irritable bowel syndrome
iNOS: Inducible nitric oxide synthase
IL-10R: IL-10 receptor
IECs: Intestinal epithelial cells
Ile: Isoleucine
Kyn: Kynurenine
Leu: Leucine
mTOR: Mechanistic target of rapamycin
Met: Methionine
MAPK: Mitogen-activated protein kinase
NEAAs: Nonessential amino acids
NF-*κ*B: Nuclear factor-kappa-B
Nrf2: Transcription factor NF-E2-related factor 2
Phe: Phenylalanine
Pro: Proline
Pm: Pasteurella multocida
PL: Poly-L-lysine
ROS: Reactive oxygen species
Ser: Serine
SERT: Serotonin reuptake transporter
Ser: Serine
SCFAs: Short chain fatty acids
Trp: Tryptophan
Thr: Threonine
Tyr: Tyrosine
Lys: Lysine
Th17: T-helper type 17
Tyr: Tyrosine
TPH-1: Tryptophan hydroxylase-1
TLR4: Toll-like receptor 4
TJPs: Tight junction proteins
UC: Ulcerative colitis
Val: Valine
5-HT: 5-Hydroxytryptophan.
